# Effect of octenyl succinic anhydride modified starch on soy protein-polyphenol binary covalently linked complexes

**DOI:** 10.3389/fnut.2023.1093250

**Published:** 2023-02-09

**Authors:** Die Dong, Tenglong Geng, Bo Cui, Chao Yuan, Li Guo, Meng Zhao, Feixue Zou, Pengfei Liu, Hongxia Zhang

**Affiliations:** State Key Laboratory of Biobased Material and Green Papermaking, Department of Food Science and Engineering, Qilu University of Technology (Shandong Academy of Sciences), Jinan, Shandong, China

**Keywords:** octenyl succinic anhydride-modified starch, EGCG (-)-epigallocatechin-3-gallate, complexes, structure, soy proteins

## Abstract

The present study aimed to investigate the effects of octenyl succinic anhydride modified starch (OSAS) on soy protein (SP)-(-)-epigallocatechin-3-gallate (EGCG) binary covalently linked complexes. Mean diameters of OSAS-SP-EGCG complexes decreased from 379.6 ± 54.9 nm to 272.7 ± 47.7 nm as the OSAS-to-SP-EGCG ratio changed from 1:2 to 4:1, while ζ-potential decreased from -19.1 ± 0.8 mV to -13.7 ± 1.2 mV. Fourier transform infrared spectroscopy results revealed that the characteristic peaks at 1725 cm^–1^ and 1569 cm^–1^ for OSAS disappeared in the OSAS-SP-EGCG complexes, indicating an interaction between OSAS and SP-EGCG complexes. X-ray diffraction analysis showed that with the increase of OSAS content, the diffraction peak at approximately 8.0° decreased from 8.22° to 7.74°, implying that the structures of OSAS and SP-EGCG complexes were rearranged after forming into OSAS-SP-EGCG complexes. The contact angle of the OSAS-SP-EGCG complexes significantly increased from 59.1° to 72.1° with the addition of OSAS increased, revealing that the addition of OSAS improved hydrophobicity of the SP-EGCG complexes. Transmission electron microscopy images revealed that the individual OSAS-SP-EGCG complexes became smaller but stuck together to form large fragments, which was different from the morphology of OSAS and SP-EGCG complexes. Thus, the OSAS-SP-EGCG complexes developed in this study may be effective emulsifiers for improving the stability of emulsion systems in the food industry.

## 1. Introduction

Proteins and polyphenols are widely found in various foods, playing important roles in food production and nutritional value (1). In food systems, the interactions between proteins and polyphenols during storage and processing cannot be avoided. Currently, protein-polyphenol complexes have received significant research interest. Proteins and polyphenols combine with phenolic hydroxyl groups and polar sites of the protein, such as hydrogen bonding and hydrophobic bonding, to form conjugates with specific functional properties (2, 3). In recent years, protein-polyphenol complexes have attracted increasing attention (4–6).

Soy protein (SP) is one of the most naturally widely used vegetable proteins and contains essential amino acids similar to those found in animal proteins (7). (-)-Epigallocatechin-3-gallate (EGCG), the major catechin in green tea, has high antioxidant activity attributed to the active phenolic hydroxyl groups and has been endowed with numerous health benefits (8). SP and EGCG can form non-covalent-linked and covalent-linked complexes via different types of interactions (9). In recent years, SP-EGCG complexes have been investigated in many studies, including their formation (9), functional properties (10, 11), antioxidant properties (12), and their use in emulsion stability and nutrient delivery (13, 14). However, it is difficult for SP-EGCG complexes to meet the different requirements of food systems. Thus, other food ingredients should be used to broaden the use of SP-EGCG complexes in the food industry. As reported previously, the addition of polysaccharides can influence the formation of SP-EGCG complexes and improve their physical and functional properties (12, 15).

Octenyl succinic anhydride-modified starch (OSAS) is produced using an esterification reaction between –OH groups on starch and the OSA reagent. OSAS has amphiphilic properties because it consists of a hydrophilic starch backbone with attached hydrophobic octenyl groups (16). The hydrophobic octenyl group impart an emulsifying capability to OSAS (17). Previous studies have shown that OSAS can be used as a food ingredient or emulsifier in food systems (18), as the OSAS molecule can form a thick coating around the oil droplets to increases the steric repulsion between the droplets (19). However, to our knowledge, there are no reports on the use of OSAS to improve the properties of SP-EGCG complexes.

The present study investigated the effects of OSAS on SP-EGCG complexes. The formation and structural properties of the OSAS-SP-EGCG complexes were characterized by mean diameter and ζ-potential measurements, Fourier transform infrared spectroscopy (FTIR), X-ray diffraction (XRD), contact angle analysis, and transmission electron microscopy (TEM). The results found here will help better understand the formation of OSAS-SP-EGCG complexes and aid in the design of new food materials for nutraceutical and biomaterial applications.

## 2. Materials and methods

### 2.1. Materials

SP were prepared as previously reported (20). The dried SP powder had 92.8% protein content, as determined by rapid N exceed (Elementar, Langenselbold, Germany), with a nitrogen conversion factor of 6.25. OSAS (CAS#66829-29-6) was purchased from Shanghai Yuanye Bio-Technology Co., Ltd. EGCG (purity ≥ 98%) was purchased from Shanghai Macklin Biochemical Co., Ltd. All chemicals used were of analytical grade and used without further purification. Distilled water from a Lichun water purification system (Lichun, Jinan, China) was used in all the experiments.

### 2.2. Preparation of SP-EGCG covalently linked complexes

SP-EGCG complexes formed by covalent interactions were prepared according to the procedures described by Ju et al. (21) with some modifications. Briefly, 2.0 g SP was dissolved in 100 mL of distilled water at 25°C, and the pH of the dispersion was adjusted to 9.0 using 0.5 mol/L NaOH. SP-EGCG complexes were formed by mixing the SP solution with an EGCG concentration of 0.2% (w/v) for 12 h at 25°C. Dialysis was performed for 24 h using distilled water to remove free EGCG from the protein dispersion. Then, the pH of the dispersion was adjusted to 7.4 to stop the reaction. The dialysate was freeze-dried for further use.

### 2.3. Preparation of OSAS-SP-EGCG complexes

Octenyl succinic anhydride-modified starch dispersions (2%, w/v) were prepared by dispersing OSAS powder in distilled water, followed by gelatinization in a water bath at 95°C for 30 min to obtain a homogeneous solution. Fresh starch solution was prepared for each experiment.

Covalently linked SP-EGCG complexes were dispersed in distilled water at a concentration of 2% (w/v). The SP-EGCG complex dispersion was then mixed with OSA-modified starch dispersions (2%) in ratios of 1:4, 1:2, 1:1, and 2:1 (w/w). Each sample was adjusted to pH 7.0 and then freeze-dried. The resultant powders were incubated at 60°C and 79% relative humidity (RH) in the presence of saturated KBr solution for 24 h (22). The samples were pre-frozen and freeze-dried for further use.

### 2.4. Mean particle diameter and surface potential analysis

The mean particle diameter and surface potential of the OSAS-SP-EGCG complexes and covalently linked SP-EGCG complexes were measured at 25°C using dynamic light scattering and electrophoresis (Nano ZS, Malvern Instruments, Worcestershire, UK). Suspensions of the OSAS-SP-EGCG complexes were diluted 10-fold using a buffer solution to obtain an appropriate light intensity for reliable measurements. The mean and standard deviation were calculated from measurements of at least three samples.

### 2.5. FTIR

Freeze-dried OSAS, SP-EGCG, and OSAS-SP-EGCG complexes were combined with KBr and ground to form a mixture, which was then molded into a disk and analyzed using an FTIR spectrometer (Nicolet iS10, Thermo-Fisher, Waltham, MA, USA) at 25°C. The scanning range was set to 400–4000 cm^–1^ with a resolution of 4 cm^–1^ and 64 scans (23).

### 2.6. XRD

XRD (Ultima IV, Rigaku, Japan) was performed to determine whether OSAS, SP-EGCG, and OSAS-SP-EGCG complexes had crystalline structures or were amorphous, using a method similar to that used by Niu et al. (24). Briefly, freeze-dried OSAS, SP-EGCG, and OSAS-SP-EGCG complexes were analyzed using Cu Kα radiation, a scan angle of 2θ, a range of 5–60°, a scan rate of 2°/min, and working conditions of 40 kV and 40 mA.

### 2.7. Contact angle measurement

The contact angles of the OSAS, SP-EGCG, and OSAS-SP-EGCG complexes were determined using an OCA 40 (Dataphysics Instruments GmbH, Stuttgart, Germany) following the method described by Dai et al. (25). First, the dried sample powders were pressed to obtain particle-based tablets (13 mm diameter and 2 mm thickness). A drop of pure water (5 μL) was then lightly dripped onto the surface of the tablets. After equilibrium was reached, the droplet was photographed, and the profile of the droplet was automatically fitted to the Laplace-Young equation using software to acquire the contact angle. Measurements were performed at least three times.

### 2.8. Transmission electron microscopy

The morphology of the OSAS-SP-EGCG complexes was determined by transmission electron microscopy (TEM). Briefly, the samples were diluted 10-fold using double-distilled water and then placed onto a copper mesh grid for 4 min. The sample was then stained using a 1% uranyl acetate solution (1 min) and washed with double-distilled water three times. The sample-loaded grid was air-dried at room temperature and imaged using a commercial TEM (JEM-2100F, JEOL, Ltd., Tokyo, Japan) operating at a voltage of 200 kV.

### 2.9. Statistical analysis

All measurements were performed in triplicate. Statistical significance (*p* < 0.05) was determined using SPSS statistical analysis program (SPSS Inc., Chicago, IL, USA). All data shown represent mean ± standard deviation (SD).

## 3. Results and discussion

### 3.1. Mean particle diameter and surface potential of OSAS-SP-EGCG complexes

**FIGURE 1:**
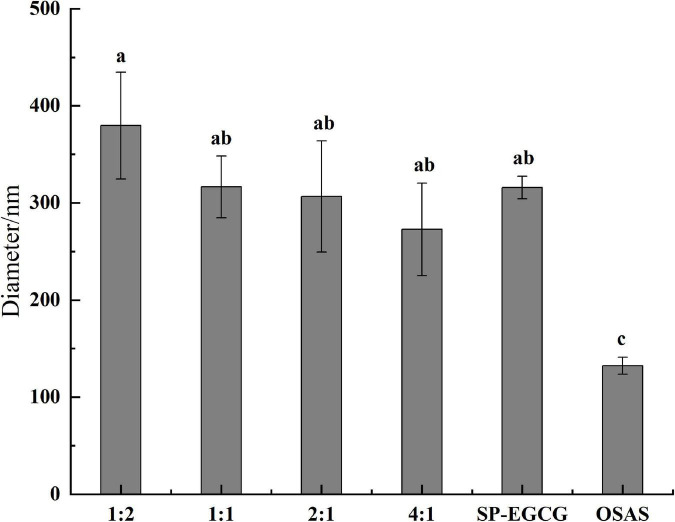
The average diameters for OSAS-SP-EGCG complexes, SP-EGCG complexes and OSAS.

The average sizes of the OSAS, SP-EGCG complexes, and OSAS-SP-EGCG complexes are shown in Figure 1. The OSAS and SP-EGCG complexes had an average particle size of 132.2 ± 8.8 nm and 315.8 ± 11.7 nm, respectively. Meanwhile, the average particle size noticeably changed to 379.6 ± 54.9 nm, 316.5 ± 31.8 nm, 306.6 ± 57.2 nm and 272.7 ± 47.7 nm for OSAS-SP-EGCG complexes with mass ratios of OSAS to SP-EGCG at 1:2, 1:1, 2:1, and 4:1, respectively. With a lower addition of OSAS (i.e., the mass ratio of 1:2), the average size of the OSAS-SP-EGCG complexes increased significantly compared with that of the OSAS and SP-EGCG complexes. One speculative possibility is that SP-EGCG was excessive under these conditions. As a result, more SP-EGCG was bound to the surface of OSAS, resulting in micro-aggregation and the formation of large composites (26). It is also possible that the higher concentration of SP-EGCG resulted in a loose structure of the OSAS-SP-EGCG complexes, which may be due to the conformational changes induced by some other interactions between OSAS and SP-EGCG. By continuously increasing the concentration of OSAS (i.e., the OSAS-to-SP-EGCG mass ratio from 1:1 to 4:1), the average particle size significantly decreased (*p* < 0.05). This may be explained by the possibility that a relatively compact structure between OSAS and SP-EGCG was formed with sufficient OSAS content under these conditions.

**FIGURE 2:**
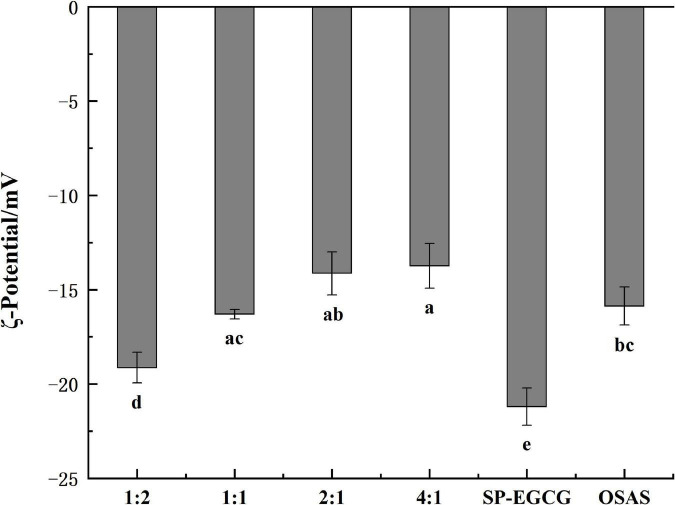
The ζ-potentials for OSAS-SP-EGCG complexes, SP-EGCG complexes and OSAS.

Figure 2 shows the surface charge densities of the OSAS, SP-EGCG complexes, and OSAS-SP-EGCG complexes. The results showed that the ξ-potentials of all samples examined were negative. The ξ-potentials for OSAS and SP-EGCG complexes were -15.9 ± 1.0mV and -21.2 ± 1.0 mV, respectively. The result corresponded to those of Zhao et al. (15) and Liu et al. (27), which were −16.8 mV and −18.0 mV for OSAS and SP-EGCG complexes, respectively. The OSAS-SP-EGCG complexes showed lower absolute ζ-potential values than that of the SP-EGCG complexes. This may be because the added OSAS interacted with the SP-EGCG complexes, causing a reduction in the net charge on the surface of OSAS-SP-EGCG, resulting in a reduction in ξ-potentials. With the mass ratio of OSAS to SP-EGCG complexes from 1:2 to 4:1, the absolute values of ξ-potentials for OSAS-SP-EGCG complexes continued to decrease from −19.1 ± 0.8 mV to −13.7 ± 1.2 mV. This may be the reason for the interaction between the -COO^–^ of OSAS and -NH_3_^+^ of SP-EGCG complexes. With the increase in OSAS content, more -NH_3_^+^ groups of SP-EGCG complexes reacted, resulting in a reduction in the absolute values of the ξ-potentials.

### 3.2. Structure of OSAS-SP-EGCG complexes revealed by FTIR analysis

**FIGURE 3:**
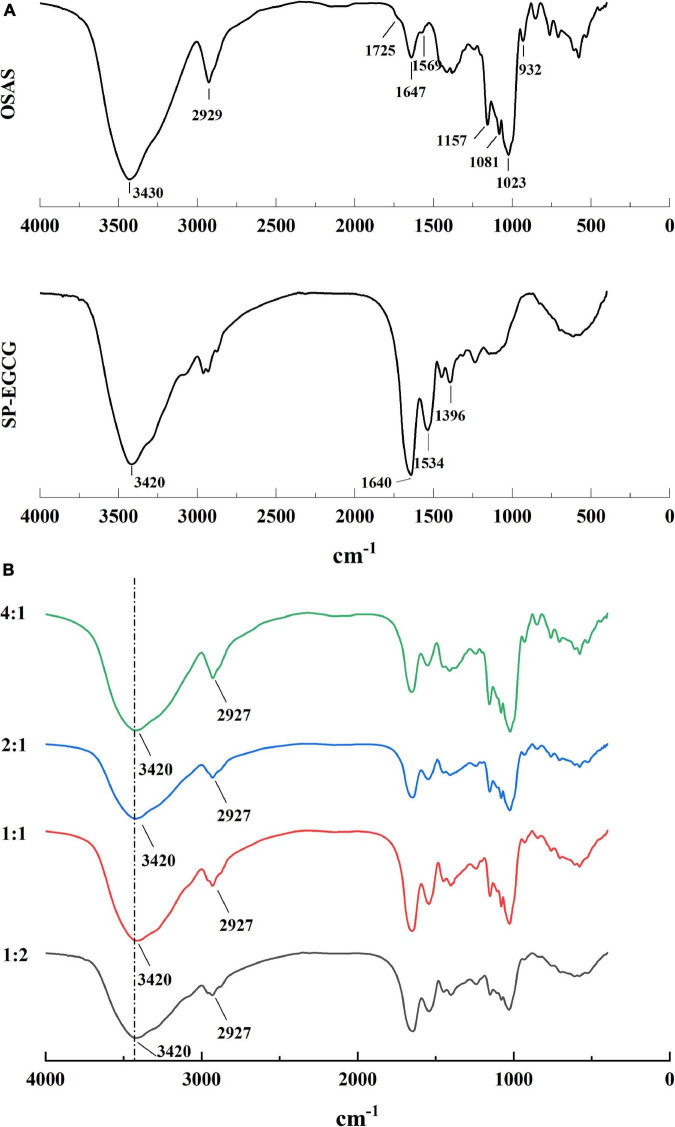
FTIR spectrum for OSAS, SP-EGCG complexes **(A)** and OSAS-SP-EGCG complexes **(B)**.

The OSAS, SP-EGCG complexes, and OSAS-SP-EGCG complexes were freeze-dried, and the interaction between OSAS and SP-EGCG complexes was characterized by FTIR (Figure 3).

For both the OSAS and SP-EGCG complexes, there was a broad peak at approximately 3,400 cm^–1^, which can be attributed to O–H stretching vibrations. For OSAS, characteristic peaks were also observed at 1,725 cm^–1^, 1,569 cm^–1^, 900–1,200 cm^–1^, 2,929 cm^–1^, and 1,647 cm^–1^. The characteristic peak at 1,725 cm^–1^ was assigned to the C = O stretching vibration of the ester group, whereas the peak at 1,569 cm^–1^ was ascribed to the asymmetric stretching vibration of the carboxyl group (28, 29). The peaks at 900–1,200 cm^–1^ were characteristic of polysaccharide functional groups (27). The peak at 2,929 cm^–1^ was attributed to the C-H stretching vibration of the glucose unit (30). The peak at 1,647 cm^–1^ can be attributed to the bending vibration of water, presumably because some water molecules are associated with powdered starch (31). The FTIR spectrum of the SP-EGCG complexes showed absorption bands related to C = O stretching at 1,640 cm^–1^ (free carboxyl groups) and N–H bending at 1,534 cm^–1^ (amide II) (Figure 3A). These two peaks are primary characteristic peaks for SP, as reported by Chen et al. (32). The absorption band at 1396 cm^–1^ is attributed to C–N stretching and N–H bending (amide III) vibrations (33).

In the mixed OSAS-SP-EGCG systems, the broad peak at 3,420 cm^–1^ did not move toward lower wavenumbers (i.e., no red-shift) compared to the OSAS and SP-EGCG complexes. These results suggest that hydrogen bonding may not have occurred between the OSAS molecule and SP-EGCG complexes. It can also be seen from Figure 3B that the characteristic peaks at 1,725 cm^–1^ and 1,569 cm^–1^ for OSAS disappeared in the OSAS-SP-EGCG complexes, indicating an interaction between OSAS and SP-EGCG complexes. Meanwhile, as the OSAS-to-SP-EGCG ratio increased from 1:2 to 4:1, the peak at approximately 2,927 cm^–1^ became increasingly obvious, indicating an increase in the OSAS content of the OSAS-SP-EGCG complexes.

### 3.3. XRD analysis of OSA-SP-EGCG complexes

**FIGURE 4:**
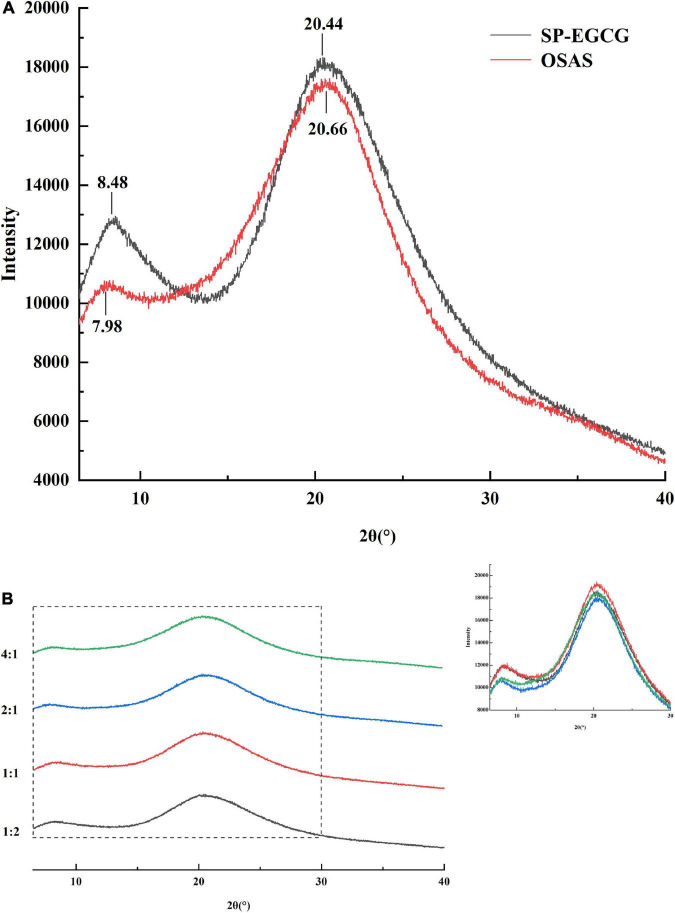
XRD spectrum for OSAS, SP-EGCG complexes **(A)** and OSAS-SP-EGCG complexes **(B)**.

XRD data can provide direct structural information for the determination of amorphous or crystalline molecular characteristics. Thus, X-ray scattering techniques are commonly used to determine the crystal structures of starches, proteins, and other macromolecular biopolymers. The main diffraction peaks of SP-EGCG complexes were located at 8.48° and 20.44° in the 2θ region, which belonged to the α-helix and β-sheet molecular structures of soy proteins, respectively (34, 35). This result agrees with the results reported by Tong et al. (6). For OSAS, the main diffraction peaks are located at 7.98° and 20.66° in the 2θ region. Previous studies found a characteristic peak at approximately 20° for V-type crystalline starch (36). As reported previously, the main diffraction peaks of OSA-modified starch are located at approximately 15°, 17°, 18°, and 23° in the 2θ region (37, 38). These differences may be due to gelatinization, after which the crystalline structures disappeared in OSAS (Figure 4A), and only two broad amorphous peaks appeared in the XRD spectra.

The OSAS-SP-EGCG complex also exhibited two peaks at approximately 8.0° and 20.5° (Figure 4B). The diffraction peak at approximately 8.0° decreased from 8.22° to 7.74° as the OSAS to SP-EGCG mixing ratio increased from 1:2 to 4:1, implying a rearrangement of the structures of the OSAS and SP-EGCG complexes after the formation of the OSAS-SP-EGCG complexes.

### 3.4. Contact angle measurement of OSAS-SP-EGCG complexes

**FIGURE 5:**
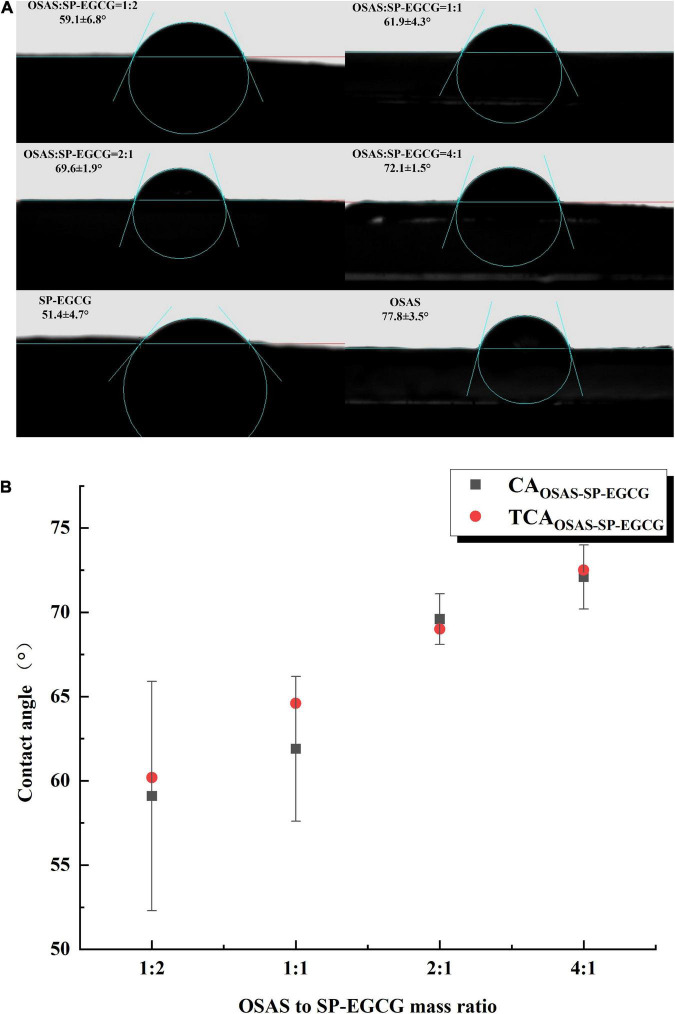
Contact angle for OSAS, SP-EGCG, and OSAS-SP-EGCG complexes **(A)** and the relationship between the measured and theoretical contact angle values for OSAS-SP-EGCG complexes **(B)**.

Water contact angle measurements enable qualitative estimation of the changes in the hydrophobicity of the samples. The water contact angle values of the OSAS, SP-EGCG complexes, and OSAS-SP-EGCG complexes are shown in Figure 5. As illustrated in Figure 5A, the contact angle of SP-EGCG complexes was 51.4 ± 4.7°, suggesting that SP-EGCG complexes were predominantly hydrophilic. The contact angle of OSAS was 77.8 ± 3.5°, indicating higher hydrophobicity. As reported previously (39), the contact angle for OSAS is related to the degree of substitution (DS) of OSA and the starch source. After the addition of OSAS, the contact angle of the OSAS-SP-EGCG complexes significantly increased from 59.1° to 72.1° as the OSAS-to-SP-EGCG mixing ratio increased from 1:2 to 4:1. The results revealed that the hydrophobicity of SP-EGCG complexes was improved by the addition of OSAS, which was beneficial for the adsorption of OSAS-SP-EGCG complexes at the oil-water interface, thereby serving as a good emulsion stabilizer.

Furthermore, the regularity of the increase in the contact angle seemed to follow the OSAS-to-SP-EGCG complex mixing ratio. To determine whether the change in contact angle could be related to the mixing ratio of OSAS to SP-EGCG complex, the theoretical contact angle value was defined as follows:


$${\text{TCA}}_{OSAS-SP-EGCG}=\;{\text{CA}}_{SP-EGCG}\;\times \;{\text{R}}_{SP-EGCG}\;+\;{\text{CA}}_{OSAS}\;\times \;{\text{R}}_{OSAS}$$

where CA_SP–EGCG_ and CA_OSAS_ are the contact angles for SP-EGCG complexes and OSAS, respectively. R_SP–EGCG_ and R_OSAS_ are the ratio of SP-EGCG complexes and OSAS in the mixtures, respectively. As shown in Figure 5B, TCA_OSAS–SP–EGCG_ for mixtures with different OSAS to SP-EGCG complex mixing ratios were in good agreement with the corresponding measured contact angles for the OSAS-SP-EGCG complexes. This indicated that the addition of OSAS greatly influenced the hydrophobicity of the SP-EGCG complexes. However, we could speculate that the actual ratio of OSAS to SP-EGCG complexes was approximately equal to the mixing ratio of OSAS to SP-EGCG complexes.

### 3.5. Morphology of the OSAS-SP-EGCG complexes

**FIGURE 6:**
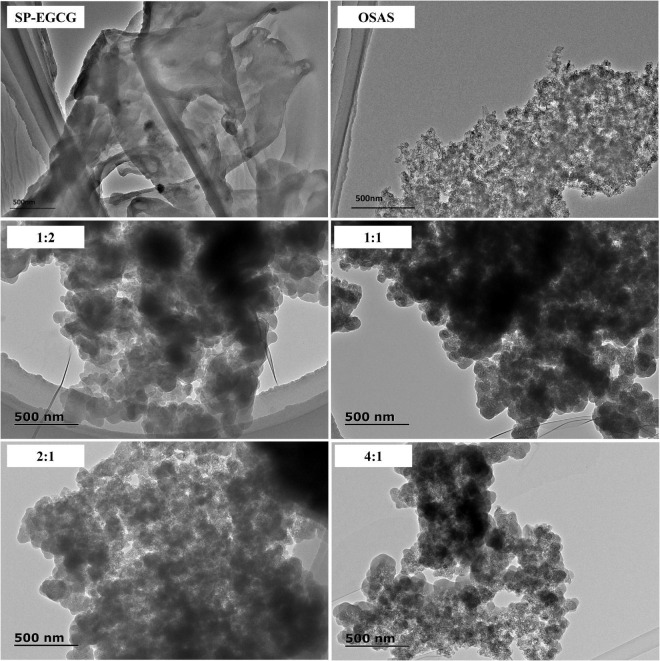
TEM images for OSAS, SP-EGCG complexes and OSAS-SP-EGCG complexes.

TEM was used to confirm the formation of OSAS-SP-EGCG complexes. Figure 6 shows the TEM images of the OSAS, SP-EGCG complexes, and OSAS-SP-EGCG complexes.

It could be seen from Figure 6 that the freeze-dried SP-EGCG complexes were distinct flaky particles with relatively larger fragments. The gelatinized OSAS were relatively smaller, and seemed to be stuck together with unsmooth surfaces. The morphologies of OSAS-SP-EGCG complexes with different OSAS to SP-EGCG mass ratios were all different from the morphology of SP-EGCG complexes. As shown in Figure 6, all the OSAS-SP-EGCG complexes were spherical with smooth surface and stuck together. This may be caused by interactions between OSAS and SP-EGCG to form tertiary complexes. With the increase in OSAS to SP-EGCG from 1:2 to 4:1, it appeared that the individual OSAS-SP-EGCG complexes became smaller but stuck together to form large fragments. This result agreed with the results showing that the mean diameter decreased as the OSAS to SP-EGCG ratio increased from 1:2 to 4:1 (Figure 1). Microscopy images indicated that the addition of OSAS had obvious effects on the microstructure of the SP-EGCG complexes.

## 4. Conclusion

In the present study, the effects of OSAS on SP-EGCG binary covalently linked complexes were investigated using diameter analysis, ζ-potential measurement, FTIR and XRD analyses, contact angle measurement, and TEM analysis. These results indicated the formation of OSAS-SP-EGCG complexes. In particular, the contact angle of the OSAS-SP-EGCG complexes prominently increased from 59.1° to 72.1° as the OSAS-to-SP-EGCG mixing ratio increased from 1:2 to 4:1. This revealed that the hydrophobicity of SP-EGCG complexes was improved by the addition of OSAS, which was beneficial for the adsorption of OSAS-SP-EGCG complexes at the oil-water interface, thereby serving as a good emulsion stabilizer. Consequently, the OSAS-SP-EGCG complexes developed here may be effective emulsifiers for improving the physical and chemical stability of emulsion systems in the food industry. In future studies, the use of OSAS-SP-EGCG complexes to stabilize oil-water emulsions should be investigated.

## Data availability statement

The raw data supporting the conclusions of this article will be made available by the authors, without undue reservation.

## Author contributions

DD and BC contributed to conception and design of the study. DD and TG organized the database. TG, CY, and LG performed the statistical analysis. DD wrote the first draft of the manuscript. MZ, FZ, PL, and HZ directed writing the manuscript. All authors contributed to manuscript revision, read, and approved the submitted version.
